# Ergonomic dual four-bar linkage knee exoskeleton for stair ascent assistance

**DOI:** 10.3389/frobt.2023.1285520

**Published:** 2023-12-06

**Authors:** Sarin Kittisares, Tohru Ide, Hiroyuki Nabae, Koichi Suzumori

**Affiliations:** Suzumori Laboratory, Department of Mechanical Engineering, School of Engineering, Tokyo Institute of Technology, Tokyo, Japan

**Keywords:** exoskeleton, wearable robot, knee joint mechanism, physical human-robot interaction, stair ascent, hydraulic artificial muscle

## Abstract

**Introduction:** Robotic exoskeletons are emerging technologies that have demonstrated their effectiveness in assisting with Activities of Daily Living. However, kinematic disparities between human and robotic joints can result in misalignment between humans and exoskeletons, leading to discomfort and potential user injuries.

**Methods:** In this paper, we present an ergonomic knee exoskeleton based on a dual four-bar linkage mechanism powered by hydraulic artificial muscles for stair ascent assistance. The device comprises two asymmetric four-bar linkage mechanisms on the medial and lateral sides to accommodate the internal rotation of the knee and address the kinematic discrepancies between these sides. A genetic algorithm was employed to optimize the parameters of the four-bar linkage mechanism to minimize misalignment between human and exoskeleton knee joints. The proposed device was evaluated through two experiments. The first experiment measured the reduction in undesired load due to misalignment, while the second experiment evaluated the device’s effectiveness in assisting stair ascent in a healthy subject.

**Results:** The experimental results indicate that the proposed device has a significantly reduced undesired load compared to the traditional revolute joint, decreasing from 14.15 N and 18.32 N to 1.88 N and 1.07 N on the medial and lateral sides, respectively. Moreover, a substantial reduction in muscle activities during stair ascent was observed, with a 55.94% reduction in surface electromyography signal.

**Discussion:** The reduced undesired load of the proposed dual four-bar linkage mechanism highlights the importance of the adopted asymmetrical design for reduced misalignment and increased comfort. Moreover, the proposed device was effective at reducing the effort required during stair ascent.

## 1 Introduction

Falling accidents are a major health risk in older people. Canadian Institute for Health Information (2011) reported that 74% of hospitalizations from major injuries for cases aged 65 and older were caused by falls. Moreover, unintentional falls are the leading external cause of death, causing 76% of deaths from injury in the 65 and older age group (Startzell et al., 2000). Most falls are unintentional (as opposed to intentional falls such as assault or suicide) and are preventable.

The specific actions involved in Activities of Daily Living (ADLs) play a crucial role in the risk of falling among older adults. Self-reported data from older adults indicate that stair ascent is one of the most challenging ADLs they encounter (Verghese et al., 2008). Statistical data also supports their concerns, with stair negotiation being some of the actions with the highest risk of accidents for the elderly (Startzell et al., 2000).

Older adults are more prone to falls due to several risk factors, including decreased muscle strength, balance impairment, and cognitive impairment (Al-Aama, 2011). The decreased muscular capability in particular is mechanically limiting older adults’ capability to perform ADLs. Hortobágyi et al. (2003) found that old adults perform stair ascents near their maximal capabilities. Another study by Böhme et al. (2022) reported a significant power deficit in the elderly during stair ascent.

The identification of stair ascent as one of the actions associated with an elevated risk of falls highlights the importance of targeting stair ascent for preventive interventions and assistive technologies. One such technology is robotic exoskeletons, which was found to be beneficial for stair ascent assistance; Woo et al. (2021) evaluated the effectiveness of a lower limb exoskeleton for stair ascent assist in healthy young persons. They found that metabolic costs, namely, net oxygen cost and total heartbeats were reduced with assistance without affecting climbing speeds. Chandrapal et al. (2013) found that a knee joint exoskeleton can significantly reduce muscle activity of an able-body user during stair ascent. These studies illustrate the promising potential of exoskeleton technology for stair ascent assistance.

However, a significant issue of current exoskeletons is physical Human-Robot Interaction (pHRI), which concerns the transmission of forces or torque between the human musculoskeletal system and the structure of the robot. The design of the wearable robot should consider ergonomics and comfort as one of its design goals (Pons, 2008). Nevertheless, the intrinsic kinematic incompatibility between human joints and robotics joints makes this a challenge.

Most lower-extremities wearable robots utilize simple revolute joints which only allow pure rotation along a fixed axis as the knee joint (Celebi et al., 2013). In contrast, while the human knee joint is functionally similar to revolute joints, its movements are much more complex; the tibiofemoral joint surface has a unique combination of rolling and sliding of the tibia on the femur (Pinskerova et al., 2000; Blaha et al., 2003; Biščević et al., 2005) due to the following reasons. First, the shape of the femoral condyle is not circular: several mathematical models have been proposed including segments of circles (Pinskerova et al., 2000), and oval (Biščević et al., 2005). Second, there are four main ligaments in the knee, namely, the Anterior Cruciate Ligament (ACL), the Posterior Cruciate Ligament (PCL), the Medial Collateral Ligament (MDL), and the Lateral Collateral Ligament (LDL). These ligaments affect the percentage of the rolling-sliding ratio of the knee. Additionally, the femoral condyle has a different shape on the medial and lateral side, resulting in an internal rotation during the flexion motion of the knee, where the femur is shown to undergo an internal rotation relative to the tibia (Kurosawa et al., 1985; Pinskerova et al., 2000; Koo and Andriacchi, 2008; Feng et al., 2016).

The mismatch between the kinematics of a wearable robot and its wearer results in an undesired misalignment. The resulting misalignment may cause discomfort from abrasion (Pons, 2008; Akiyama et al., 2012; Séguin and Doumit, 2020; Zhang et al., 2020; Asker et al., 2021; Gao et al., 2021), and unintended load which may cause injuries such as muscle damage or internal bleeding (Lee and Guo, 2010; Akiyama et al., 2012; Séguin and Doumit, 2020; Bessler-Etten et al., 2022). The discomfort caused by these devices may cause the user to abandon the equipment (Phillips and Zhao, 1993). An ergonomic knee joint mechanism based on knee biomechanics may be able to reduce these concerns.

One of the simpler mechanisms aimed to improve pHRI is a serial revolute joint (Sulzer et al., 2009). The mechanism is also present in many commercial off-the-shelf knee braces (Singer and Lamontagne, 2008). The mechanism consists of three links: thigh, knee, and shank. Two gears inside the knee part make the angle between the thigh and the knee, and the knee and the shank equal. In this mechanism, the Instant Center of Rotation (ICR) is at the center of the knee part. During knee flexion, the ICR moves backward and upwards, which differs from the J-shaped centrode of natural knees (Séguin and Doumit, 2023).

A more complex approach is the four-bar linkage, which is one of the earliest knee mechanisms utilized in prosthetic (Radcliffe, 1994). The goal of utilizing the four-bar linkage in lower limb prostheses was to increase stability and achieve knee lock in the stance phase of walking.

Four-bar linkage mechanisms have also been applied to simulate various aspects of the knee joint, including the ACL and PCL ligaments (Zavatsky and O’Connor, 1992), the shape of the condyle (Karami et al., 2004), and the centrode and axoid of the knee (Bertomeu et al., 2007; Kim et al., 2015; Gao et al., 2021). Zavatsky and O’Connor (1992) found that there is minimal change in the length of the ligaments during knee flexion. They proposed that a stiff bar model is a reasonable simplification of the human knee. Karami et al. (2004) optimized a four-bar linkage to the shape of the condyle. Lastly, various studies including Bertomeu et al. (2007), Kim et al. (2015), and Gao et al. (2021) replicated the centroid or axoid of the human knee using a four-bar linkage. They used optimization techniques such as genetic algorithm (GA) or particle swarm optimization to optimize the initial position of four-bar linkages. They found that four-bar linkage has a significant improvement over simple revolute joints and polycentric serial revolute joints (Bertomeu et al., 2007).

Although a planar four-bar linkage is an improvement over simpler mechanisms, it cannot account for the internal/external rotation of the tibia. In response, Zeng et al. (2022) proposed a 2-DoF humanoid dual cross four-bar mechanism, which not only utilizes the four-bar linkage to allow for ICR translation but also incorporates a set of adaptive roller mechanisms to allow for internal rotation. The researchers suggested that either a dual asymmetric four-bar linkage or a roller mechanism could be adopted to enable internal rotation, thus providing greater flexibility and precision in the design of exoskeletons.

The layout of the knee joint exoskeleton is also a crucial consideration in support design. A unilateral exoskeleton with soft support can exert an undesired twisting force (Wang et al., 2018; Sarkisian et al., 2020). This further contributes to misalignment and discomfort in the device. To address this issue, several solutions can be considered, such as adopting a two-side-support layout, using stiff support braces, or supporting on the anterior or posterior side of the leg. However, each solution comes with its own challenges: a two-side-support layout needs to account for the internal rotation of the knee, stiff braces may cause additional discomfort, and implementing anterior support is considerably more complex than other layouts (Wang et al., 2018).

In this study, we propose an exoskeleton based on dual four-bar linkage for stair ascent assistance. A two-side-support layout was adopted to minimize the undesired twisting force and reduce misalignment instead of lateral support. Two sets of asymmetric four-bar linkages are installed on the lateral and medial sides of the knee joint. To account for the kinematic differences and internal rotation of the knee joint, the four-bar linkages were specifically designed and optimized for the biomechanics of each side of the knee. This allows the device to overcome the limitations of previous four-bar linkage-based assistive exoskeletons. To actuate the exoskeleton, we selected the Hydraulic Artificial Muscle (HAM) as the actuator. The HAM has a very high force density and energy density compared to other types of actuators (Mori et al., 2010; Morita et al., 2018), making them suitable for wearable robotics. Furthermore, it is also very light, reducing the added burden to the user and improving the comfort of the device. Lastly, the HAM allows a remote actuation system to be adopted, improving weight distribution of the wearable device.

In wearable devices, the added weight and moment of inertia has a significant effect on the human user. In Royer and Martin (2005), the researchers attached several configurations of mass to the participant’s lower extremity to determine the effects of added weight and moment of inertia during walking. They found that increasing either the added weight or moment of inertia result in an increase in energy cost compared to a baseline setup. Another study (Liu et al., 2021) evaluated the effects of weight distribution of exoskeletons on muscle activities. They created four knee exoskeletons using identical components, with a different motor and gear box positioning. They found that muscle activities varied among weight distributions and movements. These studies highlights the significance of added weight and weight distribution of exoskeletons, and the importance of a lightweight actuator such as the HAM in wearable devices.

In our earlier work (Kittisares et al., 2020), we proposed a sit-to-stand exoskeleton based on a four-bar linkage and the HAM. The proposed device was able to generate the knee torque profile required for sit-to-stand and was effective at assisting the sit-to-stand in a human experiment. This study is an improvement of our earlier work, with a primary focus on reducing discomfort resulting from human-robot misalignment. In particular, the proposed design considers the kinematics of the knee in a three-dimensional space and facilitates internal knee rotation through a novel dual four-bar linkage design. This enhancement significantly improves the comfort of the device and enhances force transmission when compared to the single planar mechanism with stiff braces used in our earlier work. Moreover, the two-side-support layout enables a larger torque output, allowing the device to assist with stair ascent which demands significantly greater torque than sit-to-stand.

The remainder of this paper is organized as follows: Section 2 proposes and describes the requirements and design of the device. Section 3 evaluates the effectiveness of the device through experiments, and Section 4 discusses the implications of this study and concludes this paper.

## 2 Materials and methods

The proposed exoskeleton comprises two main components: the ergonomic four-bar linkage joint mechanisms and the HAM. The device incorporates dual four-bar linkages situated on the medial and lateral sides in a dual-side-support arrangement. These four-bar linkages serve multiple functions, including emulating the kinematics of the human knee joint, serving as attachment points for the HAM, and providing installation positions for connective equipment. The four-bar linkages are designed asymmetrically to accommodate the inherent asymmetry of the human knee joint.

The HAMs, in contrast, are the active component that provides assistive force to the exoskeleton. This force is transmitted to the user via the linkage mechanism and the connective equipment. The HAMs were selected as the actuator due to their high force density and back-drivability, both of which are crucial for wearable devices. Each linkage mechanism on both the medial and lateral sides is equipped with a HAM, enabling the delivery of assistive torque to both sides. This configuration helps reduce undesired twisting forces, thereby reducing human-robot misalignment and enhancing comfort.

### 2.1 Dual four-bar linkage design

Although four-bar linkage mechanisms have been shown to reduce kinematic incompatibility, one issue of using symmetrical four-bar linkages on both sides to replicate the kinematics of the knee joint is that it cannot account for internal/external rotation of the knee caused by kinematic differences between the medial and lateral sides. Therefore, the proposed four-bar linkage mechanism accounts for this asymmetry by reproducing the kinematics of the femur on the lateral and medial sides individually. As the medial-lateral translational motion of the tibia is relatively small, it is assumed to be negligible, and the three-dimensional motion of the tibia is approximated using two planar four-bar linkage mechanisms on the medial and lateral sides of the knee.

**FIGURE 1:**
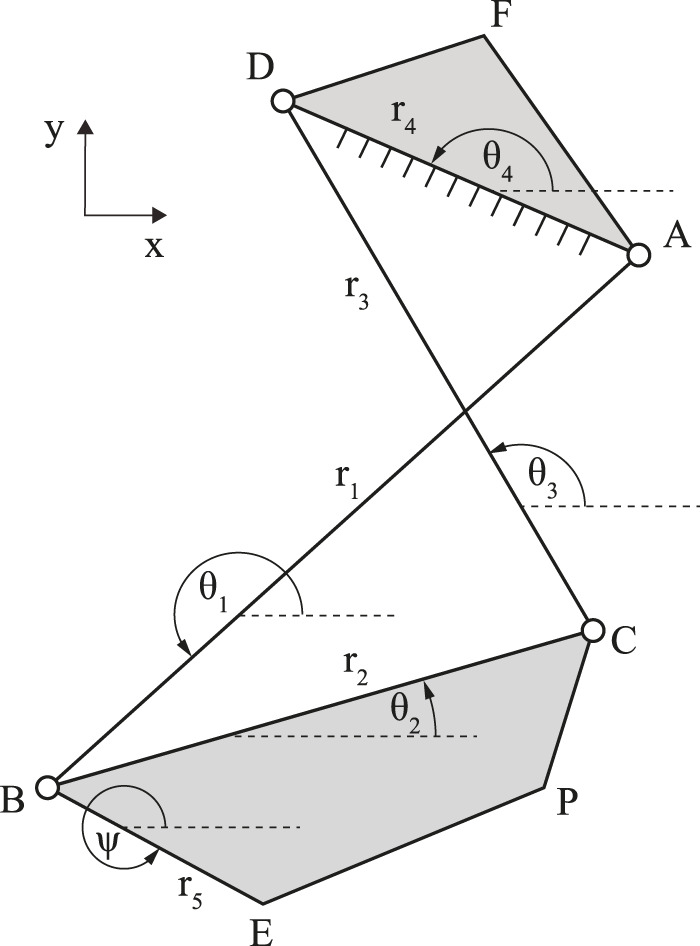
A diagram of a crossed four-bar linkage mechanism.

Consider the four-bar linkage consisting of four rigid links connected by four revolute joints with two coupler points as shown in Figure 1. The linkage mechanism was designed in moving tibia on the fixed femur coordinate, with the origin point corresponding to the geometric center axis of the condyle (Feng et al., 2016). The positive x and y values correspond to the posterior and proximal directions, respectively. Let the position of the joints be denoted by A, B, C, and D, the positions of coupler points denoted by E and P, and the angles of the links AB, BC, CD, and AD denoted by *θ*_1_, *θ*_2_, *θ*_3_, and *θ*_4_, respectively. Next, *r*_1_, *r*_2_, *r*_3_, *r*_4_, and *r*_5_ correspond to the lengths of link AB, BC, CD, AD, and BE respectively. The link AD represents the stationary femur link, while link BC represents the moving tibia. Point P denotes the coupler point which traces the path of the tibia plateau, while points E and F denote the fixing point of the HAM.

The flexion angle of the knee joint, denoted by *θ*_knee_, is defined as *θ*_knee_ = 0° when the knee is fully extended. It is given by the sum of the angle of the tibia link and its initial angle given by *θ*_*i*_:

$$\theta_{\text{knee}}=\theta_{2}+\theta_{i}.$$ (1)

The four-bar linkage mechanism can be solved using Freudenstein’s Equation (Freudenstein, 1955). Freudenstein’s Equation allows the positions of each link to be solved as a function of the angle of the coupler link. Using Freudenstein’s Equation, the relation between *θ*_1_ and *θ*_2_ is given by

$$\begin{array}{ll} k_{3}\cos\left(\theta_{2}-\theta_{4}\right)-k_{1} & =\left(\cos\left(\theta_{2}-\theta_{4}\right)-k_{2}\right)\cos\left(\theta_{1}-\theta_{4}\right)\\ & \;+\sin\left(\theta_{1}-\theta_{4}\right)\sin\left(\theta_{2}-\theta_{4}\right), \end{array}$$ (2)

where:

$k_{1}=\frac{r_{4}^{2}+r_{1}^{2}+r_{2}^{2}-r_{3}^{2}}{2r_{1}r_{2}}$ ,

$k_{2}=\frac{r_{4}}{r_{2}}$ , and

$k_{3}=\frac{r_{4}}{r_{1}}$ .

Since the angle *θ*_4_ is constant, solving the angle *θ*_1_ as a function of *θ*_2_ will allow the *θ*_knee_ to be solved. The solution of the four-bar linkage is given below.

$$\theta_{1}=atan\left(\frac{k_{4}k_{6}\pm k_{5}k_{7}}{k_{5}k_{6}\mp k_{4}k_{7}}\right)+\theta_{4}$$ (3)

where:

*k*_4_ = sin(*θ*_2_ − *θ*_4_),

*k*_5_ = cos(*θ*_2_ − *θ*_4_) − *k*_2_,

*k*_6_ = *k*_3_ cos(*θ*_2_ − *θ*_4_) − *k*_1_, and

*k*_7_ = $\sqrt{k_{4}^{2}+k_{5}^{2}-k_{6}^{2}}$ .

This will allow point B, and subsequently, points C and P, of the four-bar linkage mechanism to be found at any angle of the coupler link.

To accurately generate the trajectory of the anatomical axis using the four-bar linkage mechanism, an optimization method is necessary as it is not possible to solve for the optimal value directly. Previous research efforts focused on developing optimal ergonomic four-bar linkage mechanisms that could replicate the centrode or axoid of the anatomical knee joint. However, this approach can be prone to error, as both the tangent method and the Reuleaux method used to determine the centrode or axoid are sensitive to measurement errors (Zatsiorsky, 2002). Furthermore, small errors in the centrode or axoid can accumulate to a large final translation error due to the centrode and axoid being based on instantaneous values. Therefore, in this paper, we propose reproducing the translational motion of the tibia at any orientation instead. This will reduce the accumulative error compared to replicating the centrode or axoid.

The objective of this optimization is to minimize the error between the biomechanical trajectory of the tibia plateau and the trajectory generated by the coupler point P in the four-bar linkage mechanism. To achieve this objective, the Euclidean distance between the translational displacement of the two trajectories is minimized at any orientation. The biomechanical trajectory is based on the data published in Feng et al. (2016). The problem can be formulated as an optimization problem as:

$$\begin{array}{cc} \underset{x}{minimize}\; & \overset{\theta_{\text{max}}}{\underset{i=0}{\sum }}\left\|f\left(x,i\right)-P_{\text{knee},i}\right\|\\ \text{subject to}\; & f\left(x,i\right)\in R^{2}-50⪯x⪯50 \end{array}$$ (4)

where:

**x** denotes the optimization variable vector containing initial x-y coordinates of points A, B, C, and D, i.e., **x** = ⟨*x*_*A*_, *x*_*B*_, *x*_*C*_, *x*_*D*_, *y*_*A*_, *y*_*B*_, *y*_*C*_, *y*_*D*_⟩,

*f* denotes the function of position vector of point P of the four-bar linkage at angle *i*,

*θ*_max_ denotes the maximum knee flexion angle, and

**P**_knee,*i*_ denotes position vector of the biological knee joint at angle *i*.

The maximum and minimum values of joint positions are in place to avoid a bulky and unfeasible solution. Since the objective function of the optimization is nonconvex, a global optimization technique is required. In this study, the parameters were first optimized using GA, then the optimization results were further fine-tuned using the quasi-Newton method.

The GA is a suitable optimization method for four-bar linkage design due to its ability to solve highly nonlinear optimization problems with complex constraints. Four-bar linkage design involves determining the lengths of the links and the initial joint angles to achieve specific motion requirements. The design space for these variables can be vast and contains multiple local minima. The GA’s ability to explore a nonlinear search space allows it to search for solutions in this complex optimization problem.

The GA in this study was implemented using the Global Optimization Toolbox in MATLAB 2021a (MathWorks, MA, United States). The population size was 2000, as a smaller population was not able to explore the search space sufficiently to reach a reasonable solution due to the complexity of the four-bar linkage problem. Other options were default options, namely, the selection method was Stochastic Universal Sampling, the crossover method was Crossover Scattered, and the mutation function was Gaussian mutation.

To further optimize the results obtained from GA, a quasi-Newton method was used to fine-tune the results obtained from GA. A similar approach has been adopted in Chandra and Omlin (2007), where they used gradient descent, another gradient-based method, to fine-tune the optimized results from GA. By using a gradient-based method for fine-tuning, the optimization process can converge faster within the basin of attraction, which allows the optimal solution to be found more efficiently. In this study, the implementation of the BFGS algorithm in MATLAB 2021a was utilized. The optimization process was carried out 50 times each for both the medial and lateral link mechanisms to increase the success probability of the GA.

**FIGURE 2:**
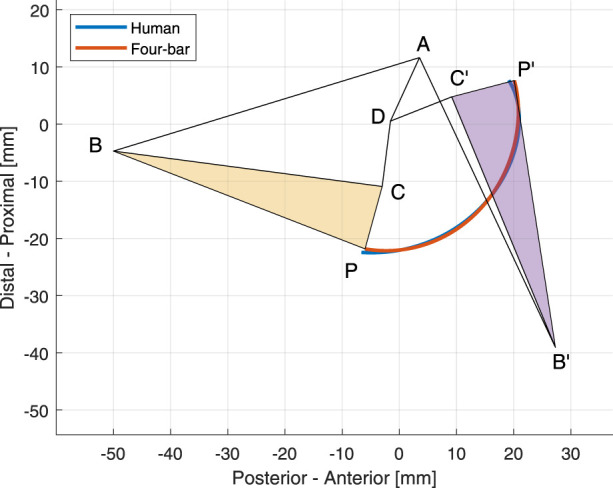
The optimal four-bar linkage configuration of the medial side at 0° and 120° knee angles. The trajectories of the human tibia and the proposed mechanism is shown in blue and orange, respectively.

**FIGURE 3:**
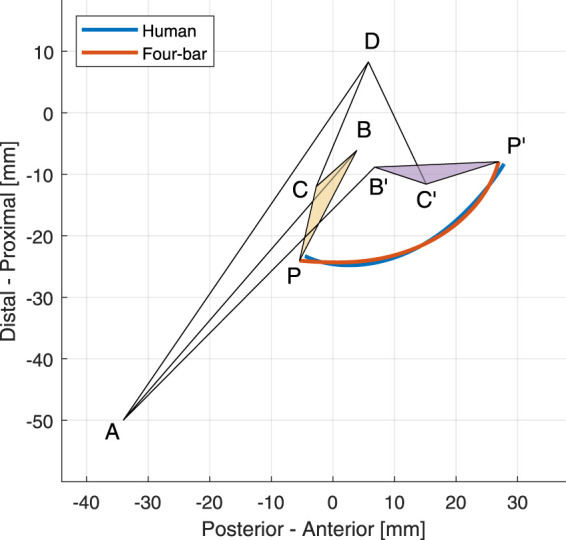
The optimal four-bar linkage configuration of the lateral side at 0° and 120° knee angles. The trajectories of the human tibia and the proposed mechanism is shown in blue and orange, respectively.

The optimization results of the medial land lateral links with the trajectory traced by the human tibia plateau and the optimal mechanism are displayed in Figures 2, 3, respectively. The initial coordinates of each point in the optimal four-bar linkage mechanisms are given in Table 1.

**TABLE 1:** Optimal x-y coordinates of the four-bar linkage in mm.

Points	Medial		Lateral	
	x	y	x	y
A	3.53	11.63	−34.05	−50.00
B	−50.00	−4.72	3.87	−6.14
C	−3.00	−10.91	−2.71	−12.01
D	−1.55	0.52	5.76	8.27

### 2.2 Hydraulic artificial muscle

**FIGURE 4:**
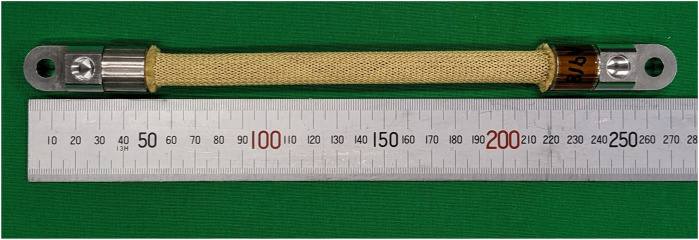
A photograph of the HAM used in this study.

The HAM, shown in Figure 4, is a contracting actuator based on the McKibben muscle. However, instead of operating with pneumatic power as originally designed (Tondu, 2012), the HAM is operated with hydraulic power. This allows it to operate at a much higher pressure, resulting in a significantly larger contraction force.

The HAM has three main components: the inner rubber tube, the outer braided sleeve, and the fittings on both ends. The outer sleeve is woven in a double-helix pattern at a specific angle. This allows the sleeve to transform the radial expansion of the inner tube into axial contraction force; when hydraulic pressure is applied to the rubber tube, it expands radially. The radial expansion of the tube, and, in turn, the sleeve, causes the braiding angle of the sleeve to widen similar to a pantograph. This phenomenon converts the radial expansion into contraction force.

The theoretical contraction force of an ideal HAM can be derived from the relationship between inflation pressure and the braiding angle of the outer sleeve. It was first given in Schulte (1961) as a function of braiding angle with correction terms for tubing elasticity and internal frictions. Ignoring the correction terms, Schulte’s equation can also be simplified as a function of contraction as

$$F=\frac{\pi {d_{0}}^{2}P}{4}\frac{1}{\sin^{2}\phi_{0}}\left(3{\left(1-\varepsilon \right)}^{2}\cos^{2}\phi_{0}-1\right),$$ (5)

where:

*F* is contraction force,

*P* is applied pressure,

*d*_0_ is initial sleeve diameter,

*ϕ*_0_ is initial braiding angle, and

*ɛ* is contraction ratio.

The theoretical maximum contraction is found at $\phi =\arctan\sqrt{2}$ or 54.74° regardless of the initial braiding angle (Tondu, 2012). This is because at this angle, the HAM has the maximum volume; contracting the HAM beyond this point produces a negative theoretical contraction force. In practice, the HAM buckles beyond maximum contraction length, and its behavior becomes unpredictable. The maximum contraction *ɛ*_max_ is given by

$$\varepsilon_{\text{max}}=1-\frac{1}{\sqrt{3}\cos\phi }.$$ (6)

The theoretical maximum contraction force is found at rest length and can be found by equating *ɛ* = 0 which results in

$$F=\frac{\pi {d_{0}}^{2}P}{4}\left(\frac{3}{\tan^{2}\phi_{0}}-\frac{1}{\sin^{2}\phi_{0}}\right).$$ (7)

However, as the simplified model completely disregards the inner rubber tube, it cannot predict the different stress-strain curves between loading and unloading of the HAM, which stems from various factors including sleeve frictions and rubber elastic hysteresis (Treloar, 2005; Tondu, 2012; Kurumaya et al., 2017). Moreover, the theoretical model greatly overpredicts the maximum contraction ratio. Therefore, the force-contraction relationship of the HAM in the design process was modeled using the least squares linear regression on the unloading curve instead.

In the human knee, the quadriceps, which is the extensor muscle of the knee, is situated on the front of the thigh. Similarly, in our exoskeleton, the HAM is connected to between link AD and BC to generate knee extension torque. The HAM is fixed to the tibia link BC at the coupler point E, and fixed to the femur link AD at point F.

The torque output of the device can be calculated from the cross product of the lever arm vector between the ICR of the four-bar linkage and the force vector. The ICR of link BC is located at the intersection between link AB and CD.

$$x_{\text{ICR}}=\frac{\left(x_{A}y_{B}-y_{A}x_{B}\right)\left(x_{C}-x_{D}\right)-\left(x_{A}-x_{B}\right)\left(x_{C}y_{D}-y_{C}x_{D}\right)}{\left(x_{A}-x_{B}\right)\left(y_{C}-y_{D}\right)-\left(y_{A}-y_{B}\right)\left(x_{C}-x_{D}\right)}$$ (8)

$$y_{\text{ICR}}=\frac{\left(x_{A}y_{B}-y_{A}x_{B}\right)\left(y_{C}-y_{D}\right)-\left(y_{A}-y_{B}\right)\left(x_{C}y_{D}-y_{C}x_{D}\right)}{\left(x_{A}-x_{B}\right)\left(y_{C}-y_{D}\right)-\left(y_{A}-y_{B}\right)\left(x_{C}-x_{D}\right)}$$ (9)

Finally, the torque output of the device, denoted by *τ*, is given by

$$\tau =F\frac{\left|\left(y_{F}-y_{E}\right)x_{\text{ICR}}-\left(x_{F}-x_{E}\right)y_{\text{ICR}}+x_{F}y_{E}-y_{F}x_{E}\right|}{\sqrt{{\left(y_{F}-y_{E}\right)}^{2}+{\left(x_{F}-x_{E}\right)}^{2}}}.$$ (10)

The design process to determine to locations of points E and F are as follows. First, the length of the HAM is given. Next, link BC was extended from point B by length *r*_5_ and angle *ψ*, creating point E, which is where the HAM is connected to link BC. Then, to match the contraction ratio of the HAM to the motion range of the device, a circle with a radius equal to the length of the HAM at maximum contraction was drawn with the point E at *θ*_knee_ = 0° as its center. Next, another circle with a radius equal to the HAM at rest length was drawn with point E at maximum flexion angle as its center. The two circles have two intersections, and the intersection on the proximal side (i.e., on the thigh) is selected as the fixing point F on link AD. This process ensures that the HAM will be at rest length at the maximum flexion angle, and fully contracted when *θ*_knee_ = 0°, effectively preventing the device from bending the knee beyond its natural range of motion.

The optimal values of *r*_5_ and *ψ* were selected by using grid search to optimize for the values with the maximum sum of knee extension torque between 40° and 70°, which is the range where the peak torque during stair ascent was reported in the literature. The values were also selected such that the device will not generate negative torque, hindering the motion at any knee joint position. The lateral link was designed with a slightly wider range of motion to account for internal rotation.

**FIGURE 5:**
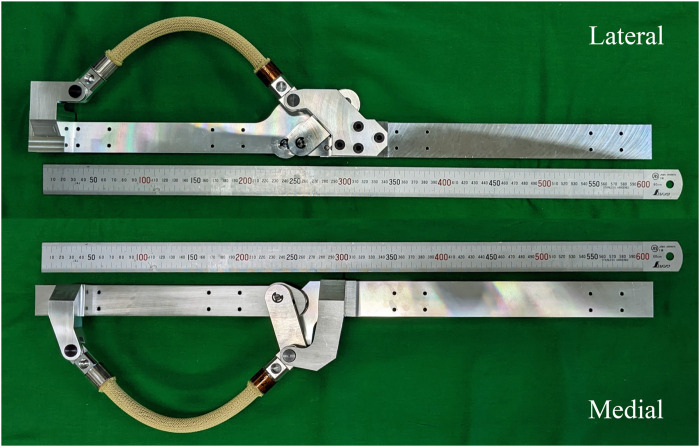
Photograph of the proposed device for the right leg on the lateral and medial sides.

The final design of the device is displayed in Figure 5.

### 2.3 Experimental validation

Two experiments were conducted to verify the effectiveness of the proposed exoskeleton. First, the improved comfort of the proposed dual four-bar linkage mechanism was evaluated using a pressure sensor to measure the unintended load acting on the leg due to human-robot misalignment. Second, a human trial has been conducted to verify the effectiveness of the device in humans. The participant in both experiments was one of the authors; therefore, ethical review and approval were not required.

#### 2.3.1 Undesired load measurement

When a wearable device with incompatible kinematics to the natural biomechanics of the human body is worn, the misalignment can cause undesired load to the user at the support equipment that connects the device to the human body. This undesired load causes discomfort and pain, and can potentially injure the user. In this experiment, this undesired load is measured to evaluate the improved comfort of the proposed mechanism.

In this experiment, stiff braces were used to connect the wearable device to the user’s body. These braces were carefully designed and 3D-printed to fit the user’s body contours, ensuring a secure and customized attachment. The braces were fixed to the user’s body using hook-and-loop fastener straps. Two braces were used for each of the shank and thigh parts of the device, resulting in a total of four braces.

**FIGURE 6:**
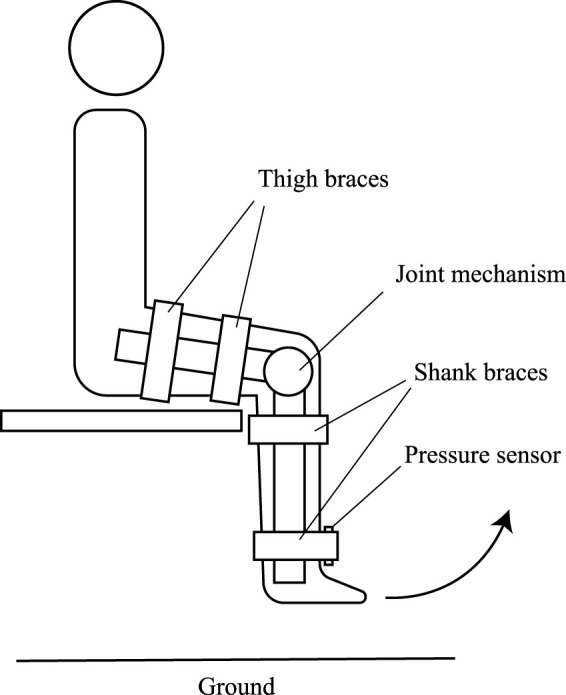
Experiment setup of the undesired load experiment. The subject was seated with their leg hanging above the ground. The pressure sensor was inserted under the lower shank brace on the anterior side of the leg.

To measure the undesired load, a thin film force sensing resistor (MD30-60, Walfront) was inserted between the lower shank brace and the user’s leg. This position is where the undesired load was felt intensely in preliminary tests. Both sides of the sensor were attached with a thin plate to normalize the contact surface. The sensor was calibrated to ensure precise sensor readings. The experiment setup is illustrated in Figure 6.

The proposed dual four-bar linkage mechanism was compared against an identical orthosis equipped with a revolute joint and a four-bar linkage proposed by Karami et al. (2004). In their paper, they concluded that the kinematic differences between the medial and lateral sides were negligible. The implementation of the Karami model four-bar linkage was discussed in detail in our previous paper (Kittisares et al., 2020). All mechanisms were outfitted with identical braces at approximately the same distance from the knee joint. The experiment was conducted on a 28-year-old healthy male with a height of 1.65 m and a body mass of 54 kg. The experimental methodology is as follows:

1. The participant wears the device while seated and the knee at approximately 90° angle.
2. The participant extends and flexes their leg a few times and readjusts the straps to align the device with the biological joint.
3. The participant is seated with their legs hanging.
4. The participant slowly extends their leg until fully extended, then slowly returns to the hanged position. This step is repeated for 10 times.

#### 2.3.2 Support evaluation

In this section, a human trial was conducted to evaluate the effectiveness of the proposed device in assisting a natural stair ascent motion in a healthy subject. The surface electromyography (sEMG) signal was monitored to measure the level of muscle activation during stair ascent with and without assistance from the device.

**FIGURE 7:**
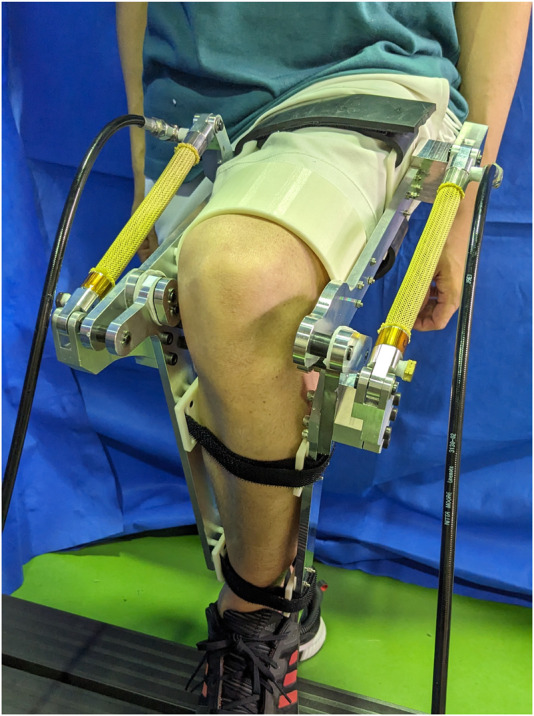
The proposed device worn by the participant.

**FIGURE 8:**
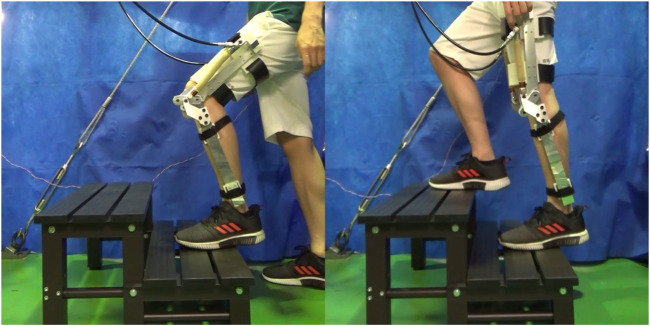
The participant performing stair ascent with the assistance of the proposed device.

The stair used in this study had two steps, with a 160 mm rise and 265 mm run. These dimensions are within the Japanese Building Standard Law. The participant was a 28-year-old healthy male with a 1.65 m body height and 54 kg body mass. The device was equipped with 3D-printed braces and slots, a rubber strap, and hook-and-loop fastener straps as shown in Figure 7. The weight of the device including connective equipment was 3.7 kg. The subject wore the device on the left leg. First, the participant placed their left foot on Step 1 in a natural position. Next, the participant climbs the stairs, lifting their right foot from the ground to Step 2 in a natural trajectory. A photograph of the participant while performing stair ascent is shown in Figure 8. The sEMG signal was measured during the stance phase of the supported leg.

A natural sEMG signal during stair ascent without wearing the device was compared with two support strategies. In the slow assistance configuration, the pump flow rate was limited to 0.58 cm^3^/sec, resulting in a gradual increase in actuator pressure. The supply pressure increased gradually similar to a ramp function until reaching the maximum pressure of 3 MPa in approximately 2 s. On the other hand, in the instant assistance configuration, the flow rate limit was increased to 2.33 cm^3^/s. This allows the supply pressure to increase to the maximum value of 3 MPa in a much shorter window. The subject performed ten trials of the experiment for each configuration.

Hydraulic pressure was supplied and controlled using a servo pump consisting of a fixed displacement water pump (ASP035-T110, LEVEX Corporation, Kyoto, Japan) and a 3-phase AC servo motor (HG-SR51B, Mitsubishi Electric Corporation, Tokyo, Japan). The pump is directly connected to the proposed device. This allows the supplied pressure to be directly controlled by varying the torque exerted by the servo motor. The servo motor was controlled using a servo amplifier (MR-J4a, Mitsubishi Electric Corporation, Tokyo, Japan) and a controller board (MicroLabBox, dSPACE Inc., MI, United States) operating at 1 kHz. The servo pump has a maximum working pressure of 5 MPa. The implementation of the hydraulic source equipment was discussed in detail in Kittisares et al. (2022), Kittisares et al. (2023).

The sEMG signal on the left Rectus femoris was measured using a surface electromyography device (FREEEMG 1000, BTS Bioengineering, MI, Italy). The signal was sampled at 1 kHz, and processed with a sliding window root-mean-square of 200 sample windows length. The signals were resampled and normalized to the stair ascent duration, and then the averages between the ten trials were calculated.

## 3 Results

### 3.1 Preliminary results

First, preliminary experiments were conducted to measure the properties of the HAM used in this study for the design process. The HAM used in this study has an initial braiding angle of 25°, an outer diameter of 12 mm and a contracting lenght of 166 mm. Other properties of the HAM used in this paper are summarized in Table 2. The HAM was initially fixed on one end, and the other end was fixed to a hydraulic cylinder rod with a load cell in between to measure the contraction force. The experimental methodology is as follows:

1. The hydraulic cylinder rod is adjusted so that the HAM is at its neutral length.
2. Hydraulic pressure is applied to the HAM.
3. The hydraulic cylinder is extended until the measured contraction force is reduced to zero.
4. The hydraulic cylinder contracts to its initial length.
5. The hydraulic cylinder is extended again until the contraction force reduces to zero.

**TABLE 2:** Properties of the HAM used in this paper.

Property	Value
Hole-hole distance	250 mm
Contracting length	166 mm
Maximum pressure	5 MPa
Maximum contraction ratio	30.2%
Outer tubing diameter	11 mm
Sleeve Diameter	12 mm
Initial braiding angle	25°
Dry weight (including fittings)	100 g

**FIGURE 9:**
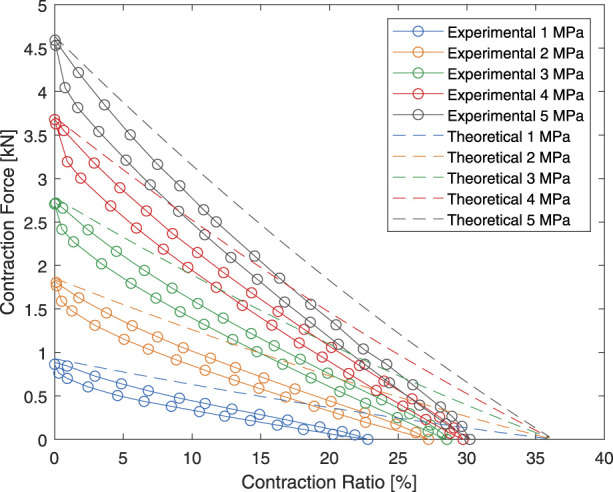
Experimental and theoretical force-contraction relationship of the HAM used in this paper.

The resulting theoretical and experimental force output are shown in Figure 9. It can be seen that the maximum contraction force predicted by the theoretical model agrees well with the experiment results especially at higher pressure, where the influences of rubber hardness and various frictions diminish in significance.

**FIGURE 10:**
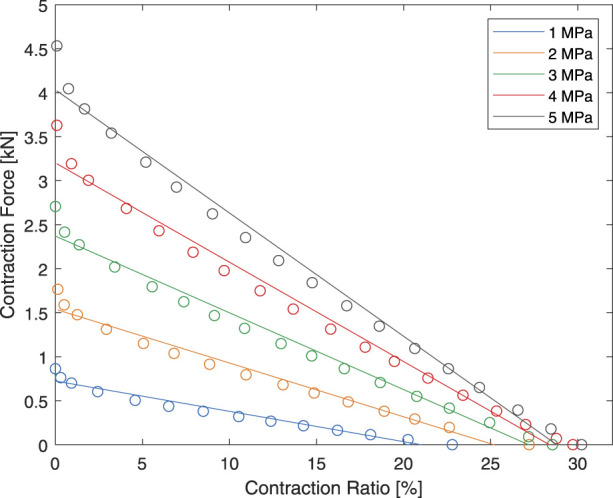
The linear model of the HAM used in the design process.

As a result, the HAM was instead modeled using linear regression for the design process. Only the unloading curve of the HAM was modeled to reduce the effects of rubber hysteresis. The linear regression model of the HAM is displayed is Figure 10.

### 3.2 Undesired load measurement

The experimental results show a significant reduction in undesired force acting on the shank part as shown in Table 3. Moreover, the participant reports significantly reduced pain at the contact area at the braces.

**TABLE 3:** The mean and standard deviation of peak load acting on the lower shank brace during extension motion in N.

Mechanism	Medial	Lateral
Revolute	14.15 (2.32)	18.32 (2.61)
Karami et al. (2004) model	10.70 (1.95)	6.48 (1.22)
Dual Four-bar	1.88 (0.26)	1.07 (0.17)

The experiment results show that in orthoses with traditional revolute joints, kinematic differences between a mechanical joint and a biological joint may cause discomfort and pain. On the other hand, the proposed four-bar linkage mechanism which aims to mimic the biomechanics of the human knee joint can significantly improve comfort by reducing undesired loads at connective equipment.

### 3.3 Support evaluation

**FIGURE 11:**
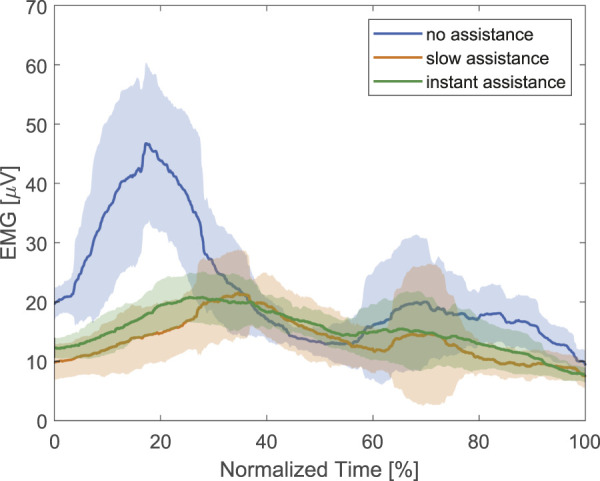
EMG signal on the Rectus femoris of the subject during stair ascent.

The processed sEMG signal showed a reduction in muscle activity with the assistance of the device as shown in Figure 11. The maximum processed sEMG signal reduced from 52.34 µV without assistance to 26.09 µV with slow assistance, and 23.06 µV with instant assistance. This corresponds to a 50.15% reduction for slow assistance and 55.94% reduction for instant assistance.

## 4 Discussion

The optimized mechanism was compared to a simple revolute joint and four-bar mechanisms proposed in the literature. The positions of all mechanisms were optimized, and the coupler link of the four-bar linkage mechanism was extended with an optimal coupler point to minimize the errors. All mechanisms were compared to the data in Feng et al. (2016). The errors are summarized in Table 4.

**TABLE 4:** RMSE of the proposed mechanism compared to previous mechanisms in the literature.

Mechanism	RMSE (mm)	
	Medial	Lateral
Revolute Joint	6.61	5.69
Zavatsky and O’Connor (1992) model	5.49	1.72
Karami et al. (2004) model	5.08	1.81
Proposed dual four-bar	0.35	0.46

Comparisons with previous four-bar linkage designs confirmed the importance of an asymmetric design to reduce human-robot misalignment. Although Karami et al. (2004) suggested that the kinematic differences between medial and lateral sides are negligible, comparisons with data reported in Feng et al. (2016) contradicts with their assumption; four-bar mechanisms in Zavatsky and O’Connor (1992) and Karami et al. (2004) showed a significantly reduced error compared to a revolute joint on the lateral side, from 5.69 mm to 1.72 mm and 1.81 mm, respectively. However, the errors were much larger on the medial side, at 5.08 and 5.49 mm, respectively. This highlights the importance of using an asymmetric four-bar linkage design, which has a much lower RMSE of 0.35 and 0.46 mm on the medial and lateral sides, respectively.

Experimental findings also supported the theoretical outcomes, as the (Karami et al., 2004) model exhibited a notably larger undesired load on the medial side. This highlights the importance of the asymmetrical design adopted in our exoskeleton for improved comfort.

The literature on the kinematics of stair ascent has seen several studies conducted, but there is a notable variation in their results. The reported peak torque normalized to body mass varies from as low as 0.58 Nm·kg^−1^ in Protopapadaki et al. (2007) to as high as 1.72 Nm·kg^−1^ in Larsen et al. (2009). The angle at which the peak torque occurred also varies between 40° and 70° (Andriacchi et al., 1980; McFadyen and Winter, 1988; Salsich et al., 2001; Riener et al., 2002; Hortobágyi et al., 2003; Reid et al., 2007; Reeves et al., 2009; Böhme et al., 2022). The maximum reported mean of peak flexion angle was 106° (Böhme et al., 2022).

One significant factor contributing to this variation is the age of participants involved in the studies. For instance, Hortobágyi et al. (2003) and Böhme et al. (2022) specifically examined the effects of participant demographics on stair ascent kinetics. The results revealed a significant difference in peak knee torque between old and young groups, highlighting the influence of participant characteristics on the observed kinetics. Other factors such as stair dimensions also impact the resulting knee torque during stair ascent.

**FIGURE 12:**
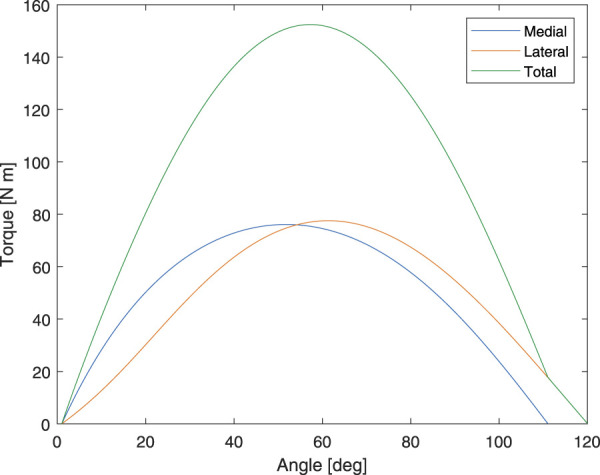
Theoretical output torque of the proposed device.

The theoretical peak torque of the proposed device is 152.44 Nm which occurs at 57°. This is equivalent to 2.09 Nm·kg^−1^ normalized to body parameters reported in De Leva (1996) and Zatsiorsky (2002). Moreover, this value is greater than the 1.72 Nm·kg^−1^ reported in Larsen et al. (2009), which is the highest value found in the literature. Therefore, we believe that the developed device is capable of providing sufficient assistive torque for stair ascent assistance. The theoretical torque output of the device on both sides and the total torque are presented in Figure 12.

A human trial was conducted to evaluate the supportive capabilities of the proposed exoskeleton. sEMG signals were used to measure muscle activity during stair ascent with and without the device, and the results showed a significant reduction in muscle activity when the device provided assistance, with the sEMG signal reducing by 55.94% from 52.34 to 23.06 µV. This reduction in muscle activity indicated that the exoskeleton effectively offloaded the user’s muscles, reducing the effort required during stair ascent.

Two assisting strategies, namely, slow assistance, which gradually increases the assistive torque, and instant assistance, which instantaneously applies the assistive torque, were examined. Although the instant assistance scheme showed a slightly lower sEMG level, a more gentle application of assistive force gives the user more time to adjust and stabilize their motion. This finding may serve as another consideration for the mechanical design and hydraulic systems in future iterations.

The exoskeleton is powered by a hydraulic remote actuation system, which requires the device to be tethered. This limits the operating range of the device to a limited area. However, the proposed mechanism design can still be adopted in areas where the limited operating range is not an issue such as rehabilitation. Alternatively, adopting a smaller hydraulic pump design (Nath and Durfee, 2017; Mao et al., 2021) or a follower robot (Tani et al., 2011) can enable the exoskeleton for outdoor use.

In summary, this study proposes an exoskeleton designed for aiding stair ascent with a primary focus on the reduction of discomfort resulting from human-robot misalignment. The device incorporates a novel dual four-bar linkage mechanism that considers the asymmetry of the medial and lateral sides of the condyle, resulting in a more anatomically accurate motion. The kinematics of the mechanism were optimized using a genetic algorithm, aiming to replicate the translational motion of the knee joint at any orientation. This optimization process helped achieve a more natural movement pattern and improved comfort.

The effectiveness of the proposed device was assessed through two experiments. The first experiment evaluated discomfort by measuring the undesired load caused by human-robot misalignment, while the second experiment assessed the device’s efficacy in a human participant. Experiment results found that the developed device exhibits significantly lower undesired loads compared to traditional revolute joint mechanisms and previous designs found in the literature. Additionally, the muscle activity of the participant during stair ascent decreased significantly with the assistance of the device.

Future work includes the improvement of the mechanical design of the mechanism and support equipment. The mechanical components of the exoskeleton could undergo further analysis and redesign to enhance their compactness, efficiency, durability, and comfort. Another area of improvement lies in the hydraulic equipment. While the experimental pump and valves used in this study confirmed the effectiveness of the proposed control schemes, actual hydraulic application necessitates a further reduction in size and weight. Lastly, conducting a human trial specifically with elderly participants would be essential to validate the exoskeleton’s effectiveness for its intended use case.

## Data Availability

The raw data supporting the conclusion of this article will be made available by the authors, without undue reservation.
